# Decline in *Clostridium difficile*-associated disease rates in Singapore public hospitals, 2006 to 2008

**DOI:** 10.1186/1756-0500-4-77

**Published:** 2011-03-23

**Authors:** Li-Yang Hsu, Thean Yen Tan, Tse Hsien Koh, Andrea L Kwa, Prabha Krishnan, Nancy W Tee, Roland Jureen

**Affiliations:** 1 Department of Medicine, National University Health System, 1E Kent Ridge Road, NUHS Tower Block Level 10, Singapore 119228, Singapore; 2 Department of Laboratory Medicine, Changi General Hospital, 2 Simei Street 3, Singapore 529889, Singapore; 3 Department of Pathology, Singapore General Hospital, Outram Road, Singapore 169608, Singapore; 4 Department of Pharmacy, Singapore General Hospital, Outram Road, Singapore 169608, Singapore; 5 Department of Laboratory Medicine, Tan Tock Seng Hospital, 11 Jalan Tan Tock Seng, Singapore 308433, Singapore; 6 Laboratory, KK Hospital, 100 Bukit Timah Road, Singapore 229889, Singapore; 7 Department of Laboratory Medicine, National University Health System, Lower Kent Ridge Road, Singapore 119074, Singapore

## Abstract

**Background:**

*Clostridium difficile *is the major cause of pseudomembranous colitis associated with antibiotic use, and the spread of the hypervirulent epidemic ribotype 027/NAP-1 strain across hospitals worldwide has re-focused attention on this nosocomial pathogen. The overall incidence and trend of *C. difficile*-associated disease (CDAD) in Singapore is unknown, and a surveillance program to determine these via formal laboratory-based reporting was established.

**Findings:**

Laboratory and pharmacy data were collated from one tertiary and two secondary hospitals on a quarterly basis between 2006 and 2008. All hospitals tested for *C. difficile *using Immunocard Toxins A&B (Meridian Bioscience Inc., Cincinnati, OH) during this period. Duplicate positive *C. difficile *results within a 14-day period were removed. The CDAD results were compared with trends in hospital-based prescription of major classes of antibiotics.

Overall CDAD incidence-density decreased from 5.16 (95%CI: 4.73 - 5.62) cases per 10,000 inpatient-days in 2006 to 2.99 (95%CI: 2.67 to 3.33) cases per 10,000 inpatient-days in 2008 (*p *< 0.001), while overall rates for *C. difficile *testing increased significantly (*p *< 0.001) within the same period. These trends were mirrored at the individual hospital level. Evaluation of antibiotic prescription data at all hospitals showed increasing use of carbapenems and fluoroquinolones, while cephalosporin and clindamycin prescription remained stable.

**Conclusions:**

Our results demonstrate a real decline of CDAD rates in three large local hospitals. The cause is unclear and is not associated with improved infection control measures or reduction in antibiotic prescription. Lack of *C. difficile *stool cultures as part of routine testing precluded determination of the decline of a major clone as a potential explanation. For more accurate epidemiological trending of CDAD and early detection of epidemic clones, data collection will have to be expanded and resources set in place for reference laboratory culture and typing.

## Background

*Clostridium difficile *is an anaerobic Gram-positive bacillus that is the major cause of pseudomembranous colitis associated with antibiotic use, accounting for 15%-25% of nosocomial cases of antibiotic-associated diarrhea [1,2]. The spread of the hypervirulent epidemic ribotype 027/NAP-1 strain across hospitals in US, Canada and Europe amidst rising incidence and mortality of *C. difficile*-associated disease (CDAD) in the past decade has re-focused attention on this successful nosocomial pathogen [1-4]. In response, the Society for Healthcare Epidemiology of America (SHEA) proposed interim standardized definitions and recommendations for CDAD surveillance in 2007 [5] and, together with the Infectious Diseases Society of America, updated clinical practice guidelines for CDAD in adults in 2010 [2].

In Singapore, the overall incidence and trend of CDAD is unknown. Data from one 1,400-bed secondary public hospital showed a rising incidence-density of CDAD from 1.49 cases per 10,000 inpatient-days in 2001 to 6.64 cases per 10,000 inpatient-days in 2006 that was coupled with a similar steep jump in the number of stool specimens tested [6]. A shorter study using a different methodology carried out over a five-month period between 2002 and 2003 at a 1,600-bed tertiary public hospital showed a CDAD point prevalence of 5.38 cases per 10,000 inpatient-days [7].

In an effort to determine the trend and incidence of CDAD locally, formal laboratory-based surveillance was conducted by the Network for Antimicrobial Resistance Surveillance (Singapore) - a voluntary group of healthcare professionals. The CDAD results were compared with trends in hospital-based prescription of major classes of antibiotics.

## Methods

A prospective surveillance program coupling laboratory-based surveillance of CDAD incidence and pharmacy-based surveillance of antibiotic prescription was implemented in five of six public sector hospitals in Singapore from January 2006 to December 2008. Two hospitals were subsequently dropped from analysis - the first because only results for 2008 were available, while the second was a specialized maternal and child hospital where CDAD rates were very low (fewer than 5 cases per quarter on average) and reliable comparative antibiotic prescription data could not be obtained because of the large pediatric population.

Hospital 1 is a 1,600-bed tertiary hospital; Hospitals 2 and 3 are secondary general hospitals with 1,400- and 900-beds respectively. *C. difficile *testing for specimens obtained at Hospital 3 is performed at Hospital 1. Both laboratories used the toxin immunoassay Immunocard Toxins A&B (Meridian Bioscience Inc., Cincinnati, OH) for *C. difficile *detection during this period. Formed stools were routinely rejected from testing.

Anonymized *C. difficile *testing data (including results, dates of testing and encoded patient identification numbers) and antibiotic prescription data were extracted from each hospital's laboratory and pharmacy electronic information systems on a quarterly basis. Denominator data in the form of hospital inpatient-days (the sum of each daily inpatient census every quarter) as well as the average length of hospitalization (LOS) for each year of the study were obtained from the hospitals' administrative records. All data were collated and analysed centrally by investigators.

Duplicate positive *C. difficile *results within a 14-day period were removed. Cumulative CDAD cases - defined as non-duplicate positive *C. difficile *stool testing [5] - and *C. difficile *testing results from all hospitalized patients were expressed as incidence-density per 10,000 inpatient-days respectively for every quarter. Antibiotics were classed as carbapenems (imipenem, meropenem and ertapenem), cephalosporins (3rd and 4th generation cephalosporins only), fluoroquinolones (ciprofloxacin, levofloxacin, moxifloxacin) and clindamycin. Defined daily dose (DDD) per 100-inpatient days for each drug prescribed every quarter was calculated following the World Health Organization (WHO) Anatomical Therapeutic Chemical (ATC) classification system 2010 [8].

Statistical analysis was performed using Stata 10.1. CDAD and *C. difficile *testing incidence-densities, and each antibiotic prescription series was tested independently for trend over time by regression analysis, corrected for autocorrelation using the Cochrane-Orcutt estimation following determination of the Durbin-Watson statistic. A coefficient of determination (R^2^) of > 0.3 coupled with *p *≤ 0.05 was considered to be a statistically significant trend result. Confidence intervals for individual incidence-densities were generated using the Poisson method.

## Results

**Figure 1:**
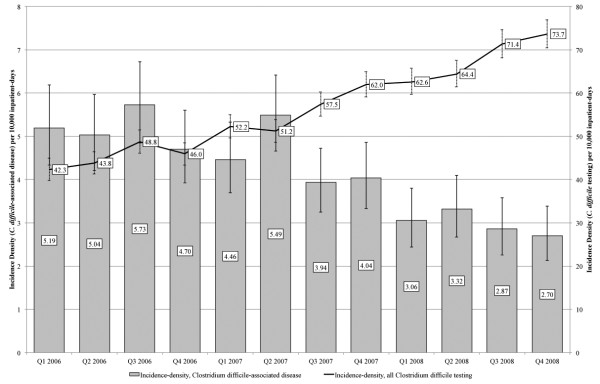
**Quarterly overall incidence-density of CDAD and *C. difficile *testing in three Singapore hospitals**.

Overall CDAD incidence-density decreased from 5.16 (95%CI: 4.73 - 5.62) cases per 10,000 inpatient-days in 2006 to 2.99 (95%CI: 2.67 to 3.33) cases per 10,000 inpatient-days in 2008 (Coefficient = -0.28; 95%CI:-0.36 to -0.20; R^2 ^= 0.87; *p *< 0.001), while overall rates for *C. difficile *testing increased from 45.27 (95%CI: 43.98 to 46.58) to 68.04 (95%CI: 66.50 to 69.61) tests per 10,000 inpatient-days within the same period (Coefficient = 2.95; 95%CI:2.54 to 3.35; R^2 ^= 0.97; *p *< 0.001). Quarterly results highlight the gradual changes over time (Figure 1). These trends are generally mirrored at the individual hospital level (Additional file 1), although Hospital 3 had markedly lower CDAD rates compared to the other two hospitals, while Hospital 2 had the highest CDAD and *C. difficile *testing rates.

Evaluation of antibiotic prescription data at all hospitals showed increasing use of carbapenems and fluoroquinolones, while cephalosporin and clindamycin prescription remained stable over the three-year period (Additional file 1). In terms of DDD/100 inpatient-days, Hospital 1 prescribed the largest relative amount of cephalosporins while Hospital 3 prescribed the largest amount of fluoroquinolones. There was a steep increase in carbapenem usage in Hospital 2, from a DDD of 2.76/100 inpatient-days in 2006 to 4.88/100 inpatient-days in 2008.

There was no significant decrease in the average LOS (range: 5.2 to 6.1 days, *p *= 0.59) over the study period at all hospitals.

## Discussion

This is the first cross-institutional surveillance on CDAD in Singapore. Our results showed a sustained decline in the incidence-density of CDAD over three years that was most marked in the two larger hospitals. This is a stark reversal from the work of Lim and colleagues, who had shown rising rates of CDAD at their institution (Hospital 2 in our study) in the few years immediately preceding the start of our surveillance program [6], whereas by 2008, CDAD rates in Hospital 2 had dropped by more than 50% back down to pre-2004 levels.

The cause of this decline is not obvious. During this same period, the prescription of key classes of antibiotics associated with CDAD did not decrease correspondingly, and the laboratory methods for *C. difficile *detection did not change. The rate of *C. difficile *testing has also increased significantly over time. While this may reflect a rising incidence of nosocomial diarrhea, it is plausible that this is the effect of increasing clinician awareness of CDAD. A general improvement of infection control and hospital hygiene could possibly have accounted for this trend, but there was no similar decline in the incidence-density of methicillin-resistant *Staphylococcus aureus *- a nosocomial pathogen whose spread is correspondingly sensitive to improved infection control standards - infections during this period using similar methodology (data not shown). There was no significant change in the average LOS over the study period at all hospitals, connoting no significant change in the mean duration of exposure for each hospitalised patient to *C. difficile*.

Koh *et al *showed that 65.6% of their institution's (Hospital 1 in our study) isolates in 2002-2003 belonged to just 3 non-027 ribotypes [6]. The decline of any of these major ribotypes - assuming these were the major *C. difficile *strains circulating in local hospitals - might account for the phenomenon presented. Unfortunately, this remains purely speculation, as culture-based techniques for *C. difficile *detection have not been in routine use in our hospitals since the start of 2006.

There are several limitations in our surveillance program. The use of a laboratory-based approach, while acceptable according to SHEA guidelines [5], may result in an overestimation of CDAD cases. The lack of cross-checking of individual hospital results by a reference laboratory may also have an impact on the accuracy of the CDAD rates reported. These are evident when compared to the 2006 results for Hospital 2, where our laboratory CDAD rates were higher (7.50 vs. 6.64 per 10,000 inpatient-days) compared to the clinical study performed by Lim and coworkers [6]. These issues are further complicated by the fact that our laboratories use commercial toxin immunoassay kits solely, which may result in unreliable epidemiological data as *C. difficile *rates continue to fall and the positive predictive value of these tests drop correspondingly [9]. Lack of stool cultures and reference laboratory testing may result in an unacceptable delay in recognizing epidemic clones such as the 027/NAP-1 clone should these be introduced into local hospitals. The data that is currently collated does not allow for the differentiation of community- and healthcare-acquired CDAD, nor does it enable tracking of mortality associated with CDAD.

## Conclusions

In conclusion, the results of our surveillance program demonstrate a real decline of CDAD rates in three large Singapore public hospitals. More work is required to elucidate the cause of this reduction, perhaps via culture and ribotyping of a sample of current *C. difficile *clinical isolates and comparing these with past results. In order to ensure more accurate epidemiological trending of CDAD and early detection of epidemic clones, data collection will have to be expanded and resources set in place for reference laboratory culture and typing of *C. difficile*.

## Competing interests

The authors declare that they have no competing interests.

## Authors' contributions

All authors contributed in the design of the study and the surveillance programme. LYH performed the statistical testing and wrote the manuscript. TTY, THK, AK, PK, NWT and RJ organized the data collection at the various hospitals and contributed to the editing and proofreading of the manuscript. All authors have read and approved the final manuscript.

## Supplementary Material

Additional file 1 **Table S1 - Incidence-density of CDAD and *C. difficile *testing at the individual hospital level, and overall antibiotic use by class**. Click here for file
