# Supplementary material for: Decline in Clostridium difficile-associated disease rates in Singapore public hospitals, 2006 to 2008
Source: BMC Res Notes. 2011 Mar 23;4:77. doi: 10.1186/1756-0500-4-77 (PMC3068971; doi:10.1186/1756-0500-4-77)
Supplement: Additional file 1 — Table S1 - Incidence-density of CDAD and C. difficile testing at the individual hospital level, and overall antibiotic use by class. [file 1756-0500-4-77-S1.DOC]

# Tables

## Table 1 - Incidence-density of CDAD and *C. difficile* testing at the individual hospital level, and overall antibiotic use by class.

| Site | 2006 | 2007 | 2008 | Trend analysis coefficient (95% confidence interval)* | *p*-value |
| --- | --- | --- | --- | --- | --- |
| Hospital 1  Number of inpatient-days  Number of CDAD cases  CDAD incidence-density (95% confidence interval)**  Number of *C. difficile* tests  *C. difficile* testing incidence-density (95% confidence interval)** | 456,406  217  4.75 (4.16 to 5.43)  1,798  39.39 (37.76 to 41.25) | 495,656  181  3.65 (3.16 to 4.22)  2,378  47.98 (46.10 to 49.94) | 457,613  140  3.06 (2.59 to 3.61)  2,998  65.51 (63.22 to 67.89) | -  -  -0.24 (-0.36 to -0.12)  -  2.37 (1.07 to 3.67) | -  -  0.001  -  0.003 |
| Hospital 2  Number of inpatient-days  Number of CDAD cases  CDAD incidence-density (95% confidence interval)**  Number of *C. difficile* tests  *C. difficile* testing incidence-density (95% confidence interval)** | 369,434  277  7.50 (6.67 to 8.43)  2,550  69.02 (66.41 – 71.75) | 407,097  280  6.88 (6.12 to 7.73)  3,417  83.94 (81.18 to 86.79) | 399,592  146  3.65 (3.11 to 4.30)  3,740  93.60 (90.66 to 96.63) | -  -  -0.41 (-0.72 to -0.10)  -  1.19 (0.36 to 2.01) | -  -  0.015  -  0.010 |
| Hospital 3  Number of inpatient-days  Number of CDAD cases  CDAD incidence-density (95% confidence interval)**  Number of *C. difficile* tests  *C. difficile* testing incidence-density (95% confidence interval)** | 210,931  41  1.94 (1.43 to 2.64)  345  16.36 (14.72 to 18.17) | 221,280  43  1.94 (1.44 to 2.62)  470  21.24 (19.41 to 23.25) | 227,261  38  1.67 (1.22 to 2.29)  641  28.21 (26.11 to 30.47) | -  -  -0.02 (-0.14 to 0.10)  -  2.81 (1.86 to 3.76) | -  -  0.678  -  < 0.001 |
| Overall Antibiotic Usage***  Carbapenem use, mean (range)  Cephalosporin use, mean (range)  Fluoroquinolone use, mean (range)  Clindamycin use, mean (range) | 3.28 (2.76 – 3.92)  16.08 (13.41 – 17.80)  37.35 (23.10 – 48.41)  3.44 (2.15 – 4.46) | 3.68 (2.27 – 4.17)  16.75 (12.72 – 18.32)  45.17 (37.76 – 51.23)  3.57 (2.42 – 4.67) | 3.88 (2.52 – 4.88)  15.91 (14.07 – 16.88)  52.33 (40.83 – 76.18)  3.81 (2.76 – 4.95) | 0.08 (0.01 to 0.16)  -0.10 (-0.63 to 0.43)  1.05 (0.41 to 1.70)  0.02 (-0.06 to 0.10) | 0.029  0.692  0.005  0.545 |

* Testing for trend over time was performed using regression analysis on quarterly data, corrected for autocorrelation using the Cochrane-Orcutt estimation following determination of the Durbin-Watson statistic. A negative coefficient implies decreasing trend over time, and vice versa.

** Measured per 10,000 inpatient-days

*** Measured as defined daily dose per 100 inpatient-days
